# Diagnosis and Management of Patients with Cardiac Sarcoidosis by a Regional Specialist Service

**DOI:** 10.3390/diseases14020076

**Published:** 2026-02-17

**Authors:** Rebecca Godfrey, Otto Fenske, Raj Selvaraju, Ana Frappell, Emine Cicek, Imad Mohamed Imran, Achuth Hosur, Eleonora Manca, Nitasha Singh, Susan Ellery, Victoria Parish, David Hildick-Smith, Jack McCready, Sabina Dizdarevic, Rachel Buxton-Thomas, John Silberbauer, Alexander Liu

**Affiliations:** 1 Sussex Cardiac Centre, Royal Sussex County Hospital, Brighton BN2 5BE, UKana.frappell@nhs.net (A.F.); emine.cicek3@nhs.net (E.C.); imadmohamed.imran@nhs.net (I.M.I.);; 2 Brighton and Sussex Medical School, University of Sussex, Brighton BN1 9PX, UK; 3 Department of Nuclear Medicine, Royal Sussex County Hospital, Brighton BN2 5BE, UK; a.hosur@nhs.net (A.H.); eleonora.manca@nhs.net (E.M.);; 4 Department of Respiratory Medicine, Royal Sussex County Hospital, Brighton BN2 5BE, UK

**Keywords:** cardiac sarcoidosis, multi-disciplinary team, specialist clinical service, cardiovascular magnetic resonance, FDG-PET-CT, immunosuppression, heart failure therapy, cardiac devices, ventricular arrhythmias

## Abstract

Background: Cardiac sarcoidosis (CS) is associated with potentially serious complications, including heart failure and life-threatening arrhythmias. The diagnosis and management of CS is multifaceted, requiring a multi-disciplinary team (MDT)-based approach. A new regional CS clinical service was established in Sussex County (UK) in January 2025. This service is based on a core of cardiologists working with a wider MDT, including specialists in pulmonary sarcoidosis, nuclear medicine and cardiac electrophysiology. This study assessed the clinical performance of this new service. Methods: Patients with suspected CS referred to the Sussex CS Service between January and December 2025 were included, as compared to a control cohort of patients referred for CS assessment before the service was conceived. Results: Of the 51 CS service referrals, 13 patients fulfilled the Heart Rhythm Society (HRS) criteria, all of whom were correctly diagnosed with CS, whilst only two out of seven HRS-positive control patients were correctly diagnosed. In the 38 HRS-negative CS service referrals, 8 patients (21%) were still given a clinical CS diagnosis compared to none in the HRS-negative controls. Of the 21 patients diagnosed with CS, 7 (33%) had active myocardial inflammation and 8 (38%) had LV systolic dysfunction. Where indicated, immunosuppressive and heart failure therapies were initiated in all patients. Eight CS patients (38%) underwent implantable cardioverter defibrillator implantation. No deaths or heart failure hospitalisations occurred within the first 11 months. Conclusions: An MDT-based CS service model can provide multi-faceted care to patients, without major short-term adverse outcomes. The service model replicability and long-term outcomes require further assessment.

## 1. Introduction

Sarcoidosis is a chronic inflammatory condition characterised by the presence of non-caseating granulomas in affected organs [1,2,3]. Although sarcoidosis affects the lung in 90% of cases, around a third of patients with sarcoidosis can develop cardiac involvement [1,2]. Cardiac sarcoidosis (CS) is associated with potentially serious complications, including advanced heart failure, ventricular tachyarrhythmias and high-grade atrioventricular heart block [4,5]. CS can also be first diagnosed on autopsy in patients who developed sudden cardiac death [6]. Although CS is often diagnosed after patients present with cardiac-sounding symptoms, clinically manifest CS accounts for only 5% of all patients with sarcoidosis [1,2,7,8]. A significant proportion of CS patients are clinically silent, as evidenced on cardiac imaging and post-mortem studies [1,7,8,9,10,11,12,13,14]. Therefore, timely diagnosis and management of CS patients before the development of cardiac complications is important.

There is no single clinical test capable of diagnosing cardiac sarcoidosis. The Heart Rhythm Society (HRS) consensus statement outlines two pathways for diagnosing CS [2]. Firstly, a CS diagnosis can be established histologically by detecting non-caseating granulomas in myocardial biopsy tissue, in the absence of an alternative cause [2]. Alternatively, a CS diagnosis is probable in the presence of histologically confirmed extra-cardiac sarcoidosis and at least one of several non-invasively determined criteria [2].

Owing to the limited availability of myocardial biopsy and associated procedural risks [12,15,16,17], the diagnosis of CS is often established based on a probability assessment, by a multi-disciplinary team (MDT) reaching a specialist consensus [4,18]. The MDT usually consists of cardiologists, respiratory physicians and cardiac imaging physicians with specialist experience in the diagnosis and management of CS [4,18]. The diagnosis is considered on a case-by-case basis, taking into account the clinical information, the confidence of the extra-cardiac sarcoidosis diagnosis, and features on advanced cardiac imaging [4,5,18]. These include cardiovascular magnetic resonance (CMR) and [^18^F]fluorodeoxyglucose positron emission tomography integrated with computed tomography (FDG-PET-CT) [1,2,4,5,7,19,20,21,22,23].

The clinical management of CS is multifaceted [4,23,24,25]. The major cornerstones include management of myocardial inflammation, cardiac dysfunction, ventricular arrhythmias and sudden cardiac death prevention [5,23,24,25,26]. Since sarcoidosis is a multisystem disorder, the management of CS often overlaps with the management of sarcoidosis in other organs [4,6,20,27,28,29]. In clinically stable patients, vigilant ongoing surveillance is usually necessary for the development of cardiac complications [1,2,3,7,18,30,31]. Therefore, CS management requires an MDT-based approach, including specialists not only within the cardiorespiratory disciplines but also physicians experienced in managing immunosuppressive therapies and multisystem sarcoidosis [2,20].

Owing to the requirements for specialist MDT-based input for diagnosis and management, CS clinical services are usually established in tertiary cardiorespiratory centres. In the UK, Sussex County has a combined population of around 1.7 million people, with its tertiary referral cardiorespiratory centre located at the Royal Sussex County Hospital in Brighton. In January 2025, a new regional Sussex CS service was established to offer MDT-based specialist care for the catchment population [32]. The service offers two outpatient clinics and inpatient care for patients with CS [32]. This study investigated the performance of this new regional CS service model for the diagnosis and management of patients, as compared to the care received by suspected CS patients before the service was conceived.

## 2. Materials and Methods

### 2.1. Study Patients

In this single-centre, retrospective observational study, all patients (aged 18 or over) referred to the Sussex Cardiac Sarcoidosis Service between 1 January to 1 December 2025 were included. Patients were assessed either in a CS outpatient clinic or managed as inpatients when their CS workup was first performed. Patients with evidence of extra-cardiac sarcoidosis referred for workup for suspected CS between June 2018 and July 2024 were also included as a control cohort (in a period before the CS service was conceived). The CS service referral patients were also compared with the pre-CS-service control cohort in a before–after service study.

### 2.2. Clinical Data

The clinical data of patients were collected from the electronic medical records hosted within the Royal Sussex County Hospital, Brighton. Patient demographic information, co-morbidities, regular medications, evidence of extra-cardiac sarcoidosis and details of clinical management were recorded.

### 2.3. Clinic Cardiovascular Magnetic Resonance (CMR)

Patients underwent clinical CMR scans at 1.5-Tesla (Aera, Siemens Healthineers, Erlangen, Germany, or Ingenia Ambition, Philips Healthcare, Best, The Netherlands). The clinical protocol included cines and LGE imaging, as previously described [33,34]. Cine images were acquired in long- and short-axis views. LGE images were acquired in matching long- and short-axis views, ~8 min after an intravenous bolus of gadolinium-based contrast agent (0.1 mmol/kg; Dotarem, Guerbet, France), followed by a saline flush. CMR images were analysed using commercially available software (Cvi42, Circle Cardiovascular Imaging, Calgary, AB, Canada) by experienced clinical CMR consultants. Biventricular volumes and systolic function were analysed by tracing the endocardial and epicardial contours in end-systolic and end-diastolic cine images. LGE was assessed visually.

### 2.4. Clinical FDG-PET-CT

Whole-body FDG-PET-CT images were acquired using a GE Discovery MI. Patients referred to the Sussex CS service underwent 48 h of dietary preparation before scanning, which consisted of 30 h of a low-carbohydrate diet followed by 18 h of fasting. This was to ensure adequate physiological glucose suppression. The presence or absence of active myocardial inflammation was reported by experienced clinical nuclear medicine consultants.

### 2.5. The Sussex Cardiac Sarcoidosis Service Model

The CS service core consists of a cardiologist and an associate specialist who provide outpatient and inpatient care, with expertise in advanced cardiac imaging, CS diagnosis/treatment, heart failure therapies and immunosuppressive drug management [32]. The service core works closely with pulmonary sarcoidosis specialists, nuclear cardiology specialists, cardiac devices and electrophysiology specialists [32]. This structure allows for individualised MDT discussions to take place for each new patient referral. The service also benefits from heart function specialist nurse support.

**Figure 1:**
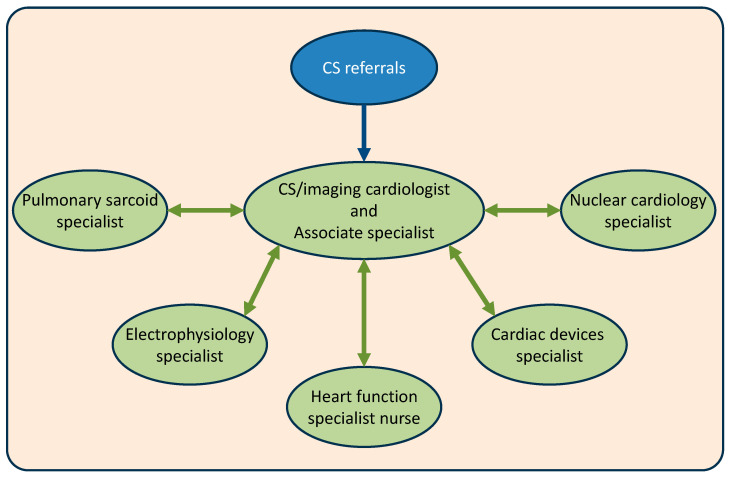
The Sussex cardiac sarcoidosis (CS) service structure.

Figure 1 illustrates the Sussex CS care structure.

### 2.6. HRS Criteria

According to the 2014 HRS Criteria, a definite diagnosis of CS is made if endomyocardial biopsy (EMB) demonstrates non-caseating granuloma with no alternative cause [2]. A probable CS diagnosis can also be made in the absence of positive EMB. This probable diagnosis requires a histological diagnosis of extracardiac sarcoidosis along with clinical or imaging findings consistent with CS [2]. The HRS criteria are detailed in Table 1. Where patients do not meet the HRS criteria, they may still be clinically adjudicated as having CS by an MDT specialist consensus in the clinical service.

### 2.7. Statistical Analysis

Data were assessed for normality using the Shapiro–Wilk test. All continuous variables further analysed statistically were parametric in this study. Parametric data were displayed as mean ± SD and compared using the independent samples Student t-test. In view of the relatively small sample sizes, categorical data were not compared using statistical tests; values were displayed to demonstrate descriptive trends. *p* values < 0.05 were considered statistically significant. Statistical analysis was performed using commercially available software (MedCalc 20.104, Mariakerke, Belgium) and validated by another observer.

### 2.8. Ethical Approval

The Research and Innovation department of the University Hospitals Sussex NHS Foundation Trust approved this retrospective study as part of a clinical service development project, and no external ethical approval was required. Informed consent from patients was waived owing to this being a retrospective study.

## 3. Results

### 3.1. Baseline Characteristics of CS Referrals

Between 1 January and 1 December 2025, a total of 51 patients with suspected cardiac sarcoidosis (mean age 58 ± 11 years; 61% males) were referred to the Sussex CS service. Of the baseline co-morbidities, hypertension (24%), heart failure (24%), hypercholesterolaemia (16%), COPD/asthma (14%), ischaemic heart disease (12%) and diabetes mellitus (10%) were amongst the most common. Of the 51 patients, 29 (57%) had evidence of extra-cardiac sarcoidosis (ECS), which was biopsy-confirmed in 15 cases.

The ECS status, co-morbidities and regular cardiac medications of the patients referred to the CS service are shown in Table 2.

### 3.2. Clinical Diagnosis of CS

Of the 51 CS referrals, 13 patients fulfilled the HRS criteria for a probable CS diagnosis, all of whom (100%) were also given a clinical diagnosis of CS by MDT assessment. Of the remaining 38 patients who did not fulfil the HRS criteria for CS diagnosis, 8 patients (21%) were clinically adjudicated as having CS by an MDT-based consensus. Of these eight patients, three patients had intense myocardial inflammation requiring urgent immunosuppressive therapy, which further supported the confidence of the CS diagnosis.

A control cohort of 24 patients with suspected CS was also included, who were referred to the general cardiology service for assessment, before the establishment of the specialist CS service (referred between June 2018 and July 2024). Seven of these patients fulfilled the HRS criteria for a diagnosis of CS.

Whilst the CS service correctly diagnosed all HRS-criteria-positive patients (13/13; 100%), only two of the seven (29%) control patients who fulfilled the HRS criteria were given a correct clinical CS diagnosis. No control patients who were HRS-negative were given a clinical CS diagnosis (vs 21% as diagnosed by the CS service).

Four patients referred to the CS service underwent EMB. Non-caseating granuloma was not isolated from any EMB samples. No control patients were referred for EMB.

The diagnostic data of the CS service referrals and controls are shown in Table 3.

The diagnostic details of the patients who did not fulfil HRS criteria but were offered an MDT-based clinical CS diagnosis are shown in Table 4.

### 3.3. Management of Myocardial Inflammation in CS Patients

Of the 51 referrals to the CS service, a total of 21 patients (41%) were given a clinical diagnosis of CS after MDT assessment. Of these CS patients, seven (33%) had evidence of active myocardial inflammation on FDG-PET-CT. Four patients received IV methylprednisolone due to the presence of intense FDG uptake in the myocardium, which completely resolved on repeat assessment with FDG-PET-CT. Two patients with myocardial inflammation received oral prednisolone and/or methotrexate therapy. One patient had severe aortic stenosis who was undergoing aortic valve surgery. An MDT decision was made to defer immunosuppressive treatment until after recovery from the cardiac surgery.

**Figure 2:**
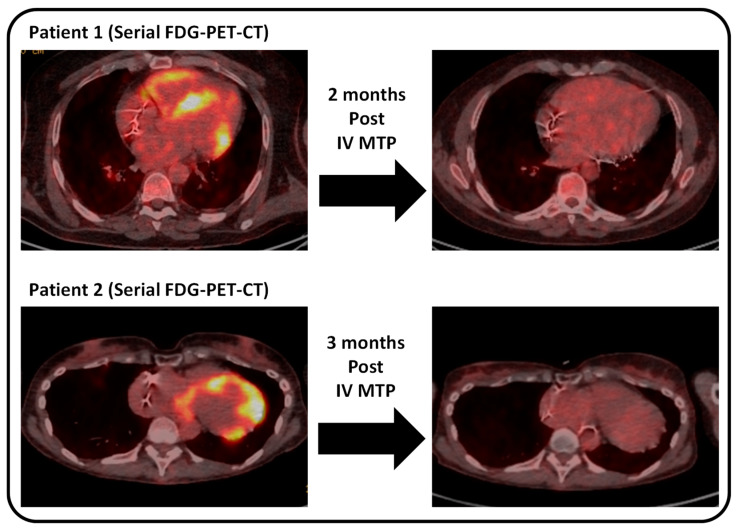
Immunosuppressive treatment response in two patients with cardiac sarcoidosis and active myocardial inflammation on [^18^F]fluorodeoxyglucose positron emission tomography integrated with computed tomography (FDG-PET-CT). Patient 1 had intense FDG uptake in the left and right ventricles, which resolved on repeat FDG-PET-CT 2 months after intravenous (IV) methylprednisolone (MTP) therapy. Patient 2 had intense FDG uptake in the left ventricle, which also resolved on repeat FDG-PET-CT 3 months after IV MTP therapy.

Figure 2 shows illustrative examples of immunosuppressive treatment responses in two patients.

### 3.4. Dietary Preparation of FDG-PET-CT Scans

Since the start of the CS service, collaborations with the nuclear medicine department have led to the introduction of a prolonged 48 h dietary preparation protocol (30 h low-carbohydrate diet followed by 18 h fasting) for cardiac FDG-PET-CT scanning. Before this collaboration, cardiac FDG-PET-CT scans were performed with 24 h dietary preparation.

Four patients scanned with the 24 h dietary preparation (before the CS service started) showed evidence of FDG uptake, and myocardial inflammation was suspected. These patients were referred to the CS service for consideration of initiation or a change in immunosuppressive therapy. All four patients were subsequently reviewed in the CS clinic, where inadequate dietary preparation was suspected on review of the FDG-PET-CT images. These patients then underwent repeat FDG-PET-CT with 48 h dietary preparation, and no FDG uptake was observed. As a result, all four patients continued their management without the need for introduction or changes in immunosuppressive therapy.

**Figure 3:**
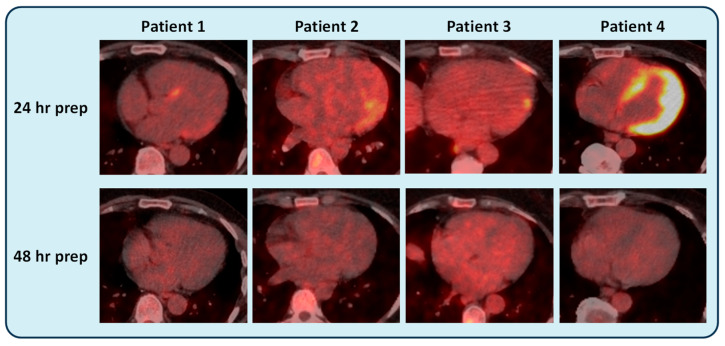
Illustrative [^18^F]fluorodeoxyglucose positron emission tomography integrated with computed tomography (FDG-PET-CT) images of CS patients with 24 or 48 h dietary preparation. Patient 1 had suspected FDG uptake in the left-ventricular (LV) septum and lateral wall on a 24 h prepared FDG-PET-CT, which disappeared on repeat scan with a prolonged 48 h preparation. Patient 2 and patient 3 both had suspected FDG uptake in the LV lateral wall on a 24 h prepared scan, which disappeared on repeat FDG-PET-CT with 48 h preparation. Patient 4 had intense diffuse global FDG uptake despite undergoing a pre-scan 24 h dietary preparation, which disappeared completely on a repeat 48 h prepared FDG-PET-CT. All 4 patients were referred for consideration of introduction or a change in immunosuppressive therapy, which were not required based on the 48 h prepared scan.

Figure 3 shows illustrative FDG-PET-CT images of the four patients with the 24 and 48 h dietary preparation protocols.

### 3.5. Management of LV Dysfunction and Ventricular Arrhythmias in CS Patients

On CMR, patients who were given a clinical diagnosis of CS had a mean LVEF of 51 ± 11% and a mean RVEF of 50 ± 12%. Late gadolinium enhancement (LGE) was present in 20/21 CS patients. Mid-wall LGE was the most prevalent (12/20 cases). Subendocardial/transmural LGE was seen in 4/20 cases. Two cases demonstrated the “shepherd crook sign” with prominent LGE distribution in both the LV and RV. One case demonstrated predominantly subepicardial LGE distribution.

**Figure 4:**
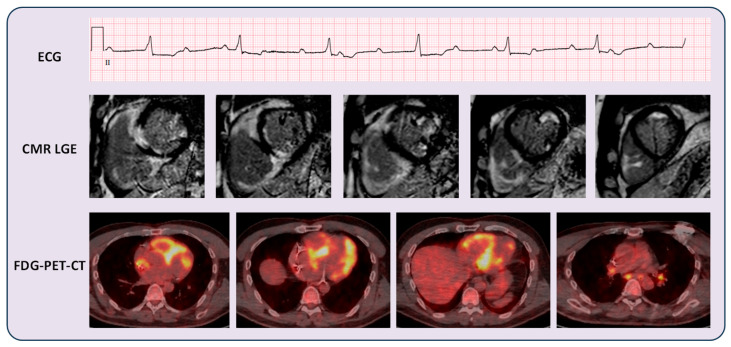
Illustrative case of a CS patient. A 56-year-old male presented with third-degree atrioventricular heart block, as shown in the 12-lead electrocardiogram (ECG, top image; lead II). His cardiovascular magnetic resonance (CMR) images demonstrated a high suspicion for cardiac sarcoidosis (illustrative late gadolinium enhancement [LGE] images shown in the second row). He underwent a cardiac resynchronisation therapy defibrillator (CRT-D) implantation. Subsequent [^18^F]fluorodeoxyglucose positron emission tomography integrated with computed tomography (FDG-PET-CT) scan showed intense biventricular FDG uptake (third row). FDG-PET-CT also demonstrated mediastinal lymphadenopathy with FDG uptake (third row, fourth image to the right). Workup did not indicate the presence of an alternative diagnosis such as lymphoma. This patient received intravenous methylprednisolone therapy and is under clinical follow-up.

Figure 4 shows illustrative images of a patient with CS.

Of the 21 CS patients, 8 (38%) had evidence of LV systolic dysfunction, defined as an LV ejection fraction (LVEF) of <50% on CMR. Seven out of the eight patients with LV systolic dysfunction were treated with optimal guideline-directed heart failure medical therapy (GDMT). These included ACEi/ARB/ARNI, beta-blockers, mineralocorticoid receptor antagonist and SGLT2-inhibitor [35]. One patient could not have GDMT due to the presence of severe aortic stenosis.

**Figure 5:**
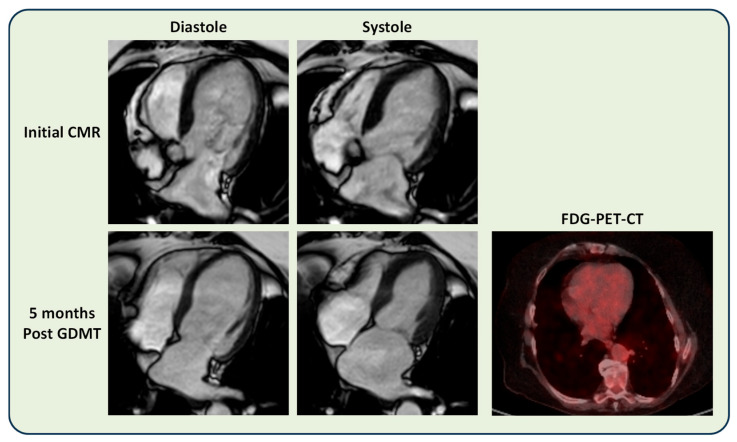
An illustrative CS patient with left-ventricular (LV) systolic functional recovery. This 67-year-old female CS patient demonstrated severe LV systolic dysfunction on her initial cardiovascular magnetic resonance (CMR) scan at workup (top row). She was commenced on guideline-directed heart failure medical therapy (GDMT), which was uptitrated to optimal doses by the heart function specialist nursing team. Five months later, her repeat CMR showed significant improvement and normalisation in LV systolic function (second row, first 2 images to the left). Her FDG-PET-CT scan did not demonstrate significant FDG uptake (second row, third image to the right).

Figure 5 shows illustrative CMR and FDG-PET-CT images of a patient who demonstrated significant LV functional recovery after a period of GDMT.

Of the 21 CS patients, 8 (38%) underwent implantable cardioverter defibrillator (ICD) implantation (n = 5 due to high-grade atrioventricular block; n = 2 due to sustained VT; and n = 1 due to LVEF < 35% despite optimal GDMT). Three patients developed further sustained VT episodes after ICD implantation and anti-arrhythmic medical therapy but without evidence of myocardial inflammation on FDG-PET-CT. These patients were referred for VT ablation procedures.

During the first 11 months of the CS service, no deaths or decompensated heart failure hospitalisations were reported.

Table 5 summarises the treatments received by the clinically diagnosed CS patients for LV dysfunction and ventricular arrhythmias.

## 4. Discussion

This study described the performance of a new regional specialist cardiac sarcoidosis service based on an MDT care model for the diagnosis and management of suspected CS patients. The main findings are: (i) the CS service could diagnose all HRS-criteria-positive CS patients, whilst only a minority of HRS-criteria-positive patients managed under general cardiology services received a CS diagnosis; (ii) in patients who did not fulfil the HRS criteria, CS was still diagnosed in around 20% of patients by an MDT-based assessment, leading to further management, including immunosuppressive therapies for myocardial inflammation; (iii) a dedicated MDT-based CS service can offer multi-faceted care to patients with no adverse outcomes in the first 11 months.

This regional specialist care structure, centred around a core of cardiologists that integrate care from different specialities, may be a resource-effective model for delivering MDT-based, multidimensional care to CS patients. The replicability of this service model in other regions may be assessed in the future to improve access to specialist care for local patients with niche conditions.

### 4.1. Diagnosis of CS Requires an MDT-Based Specialist Approach

The diagnostic challenge of CS is well-known in clinical practice, and the condition often mimics or overlaps with other cardiomyopathies [5]. Establishing an accurate diagnosis promptly, soon after clinical presentation, has an important prognostic impact on CS patients [6,8,20,21,22,26,36,37,38,39].

Despite the technical evolutions of EMB, obtaining a definitive diagnosis using EMB remains limited to selected cases, owing to variations in procedural availability and potential risks [12,17]. EMB is often reserved for cases where results from non-invasive modalities remain inconclusive, and the clinical need to reach a CS diagnosis outweighs the procedural risks of an EMB [12,15,17,38]. In this study, four patients who underwent EMB all had negative results for CS, which likely highlights the patchy nature of the condition and the contemporary low sensitivity of EMB [1,4,7]. Further work is needed to improve the safety profile, clinical availability, and targeted diagnostic yield of EMB, which is required to bring this technique to the forefront of the diagnostic algorithm for CS. Ultimately, the refinement of EMB as a technique could improve diagnostic certainty for patients with suspected CS and enable more patients to receive a definitive diagnosis.

Without definitive myocardial tissue confirmation, the diagnosis of CS remains at best probable according to HRS criteria [2]. The diagnostic criteria rely on confirming the histological diagnosis of extra-cardiac sarcoidosis (ECS) and finding other non-invasive imaging or therapeutic features that increase the likelihood of cardiac involvement [2]. This diagnostic method, therefore, hinges on estimating the probability of a patient having CS rather than arriving at a binary (yes/no) diagnosis [2]. The HRS criteria in clinical practice are important to set a clear standard for cases where a clinician should strongly consider the implications of a probable CS diagnosis [2]. However, a rigid framework of diagnostic criteria may not apply to all cases in practice, and patients who do not fulfil the HRS criteria can sometimes still be clinically adjudicated as having CS after specialist MDT assessment.

The importance of an MDT-based approach to CS diagnosis has been highlighted in several publications and institutions [1,4,5,7,18]. The diagnostic process for CS requires the adjudication of ECS. In cases where ECS is not biopsy-confirmed, the diagnostic confidence of radiological ECS requires verification by a specialist radiologist or a pulmonary sarcoidosis specialist. Alternative diagnoses, such as lymphoma, need to be ruled out as much as possible before a confident sarcoidosis diagnosis is supported [40]. The MDT-based diagnosis further considers the likelihood of cardiac involvement from advanced cardiac imaging and the response to treatment.

As seen in the patient example in Figure 4, who had patchy myocardial inflammation on FDG-PET-CT, CMR appearances compatible with CS, and presentation of high-grade atrioventricular heart block, a clinical diagnosis of CS was made after MDT assessment. This diagnosis was supported by the evidence of radiological features of mediastinal lymphadenopathy and ECS, as adjudicated by radiology specialists.

### 4.2. Specialist MDT-Based Service Improves Clinical Diagnosis of CS

Existing opinions recommend that CS patients should be managed by dedicated specialist services [4,18,22]. The data in this study indicate that more patients can be diagnosed with CS in a regional specialist service. In patients who fulfilled the HRS criteria, all were correctly given a CS diagnosis by the service, as compared to only a minority of those patients referred to general cardiology services. This reflects the need for CS patients to be diagnosed by an MDT-based clinical structure. An analogy may be that a patient with an adult congenital heart disease could be better managed under a dedicated service, rather than under general services, for access to the necessary specialist expertise and care. This intrinsic difference in specialist care availability may also explain why patients who did not fulfil HRS criteria for CS could still be given a clinical CS diagnosis by the Sussex service; none of these patients were diagnosed when under general cardiology services. The results of the study indicate that patients with suspected CS should be referred early to a dedicated service to benefit from the diagnostic implications. This recommendation concurs with existing evidence that early diagnosis of CS patients may lead to a better prognosis [26], including patients with implanted cardiac devices, if preserved LV function is maintained [24].

### 4.3. CS Management Necessitates a Dedicated MDT-Based Service

An MDT-based approach to the management of CS patients has several advantages. Firstly, CS patients can suffer a range of different cardiac complications and thus require multi-dimensional considerations in clinical practice [2]. Not only could a CS patient suffer advanced heart failure, ventricular arrhythmias, myocardial inflammation and high-grade conduction defects, but each of these complications could be connected [2]. This makes it even more important to provide comprehensive care to CS patients [4,18]. Secondly, when a CS patient experiences a cardiac complication, there may be more than one underlying aetiology, which requires an MDT-based consideration [4]. For instance, ventricular arrhythmias (VAs) may be driven by myocardial inflammation, myocardial fibrosis or both [2,4,30,41]. This is in addition to the conventional causes of VA, such as ischaemic heart disease, which also need to be considered [42]. Destination therapies for VA, such as ablation procedures, require consideration of the myocardial inflammatory status of the CS patient [1,2,7,30]; these decisions may be best determined by the MDT. Thirdly, since CS is a condition that could “wax and wane”, establishing cardiac inflammatory, electrical and functional stability does not necessarily signify the end of patient management [4]. CS patients require a carefully coordinated follow-up strategy by specialists skilled in identifying the multifaceted signs and triggers of disease flare. For this reason, it is usually recommended for CS patients to be under the follow-up of a dedicated specialist service [4].

The data in this study suggest that the newly diagnosed CS patients could receive multifaceted management of myocardial inflammation, heart failure, VA and high-grade atrioventricular heart block. No deaths or heart failure hospitalisations were reported in the patients in the short term, and further work is required to assess the long-term outcomes in the CS patients.

### 4.4. Regional Specialist Care for the Local Population

Specialist services for CS tend to be located in specific centres in the UK and worldwide. The availability of MDT expertise, auxiliary clinical services and enthusiasm of clinical leaders in supporting such a service often help to dictate whether a CS service can be established. In regions with relatively large populations, local patients may be referred for assessment to centres many miles away from their homes. Considering variations in patients’ abilities to attend clinics, investigations and treatments in far geographical locations, the lack of a regional service may contribute to healthcare inequality. This may be a particularly difficult issue for patients who find it challenging to benefit from distance clinical services.

The rationale for the establishment of the Sussex CS service hinged significantly on providing access to specialist care for local/regional patients. Ongoing work to assess patient satisfaction in surveys will further help to assess the success of the service. The suggestions from the surveys will be used to make adjustments so that the service directly listens to the patients it serves.

The design of the care structure was aimed to provide flexibility to the assessment and management of patients. The core clinicians are given the freedom to dictate which members of the wider MDT to discuss each new referral with. The consensus reached by the MDT is therefore based on the appropriate specialist opinions required to reach a diagnosis or to plan treatment. This structure makes the service relatively easy to replicate if given the necessary support as received in Sussex.

### 4.5. Limitations and Future Direction

This is a single-centre retrospective study assessing the performance of a new regional CS service. The number of patients is relatively small, which is likely vulnerable to sampling bias. With the relatively small sample size, the statistical results should also be interpreted as guidance of trends rather than definitive relationships. Future assessment of the service after more patients have been diagnosed and managed for CS will be helpful to inform on long-term performance. The CS service patient cohort was compared to a historical control cohort. While this design may be understandable in the context of service evaluation, it inherently limits causal inference. The control cohort spans a long pre-service period, during which substantial changes may have occurred in clinical awareness, imaging availability, and guideline recommendations. Therefore, the study results are best interpreted as descriptive and hypothesis-generating. Further larger studies are required to test the findings further.

Direct comparison of the treatment of CS patients in the service and the control cohort was not meaningful due to the small number of control patients who were given a CS diagnosis and variations in the clinical pathways involved in their care. The clinical service model has not been replicated in other centres, which may be considered in the future. The exact prevalence of CS in the UK Sussex County population remains unknown. As the clinical service evolves, and with the advent of further service evaluation and research, this question may become clearer.

With the development of this new service, the number of referrals is expected to increase in the future. Clear communication, effective triaging, and establishment of unambiguous referral pathways are some factors that could help to ensure the maximal effectiveness of a cost-effective service.

## 5. Conclusions

A new regional cardiac sarcoidosis service was associated with increased diagnosis of patients with CS, emphasising the value of referring suspected CS patients for dedicated specialist care. An MDT-based care model centred on a core of clinicians that fanned out to involve a wider range of specialities provided multi-faceted care to CS patients without major adverse outcomes in the short term. Further follow-up will determine the long-term outcomes within the service and the replicability of this care structure.

## Figures and Tables

**Table 1:** Focused summary of the HRS criteria for cardiac sarcoidosis (CS) diagnosis [2].

Definite CS	Probable CS
Histological diagnosis from myocardial tissue (no alternative cause)	Histological diagnosis of extra-cardiac sarcoidosis AND 1 of: Immunosuppressant-responsive heart block or cardiomyopathy Unexplained LV systolic dysfunction (LVEF < 40%) Unexplained sustained ventricular tachycardia Unexplained high-degree atrioventricular heart block FDG-PET-CT uptake consistent with CS CMR LGE consistent with CS Positive gallium uptake consistent with CS and other cause excluded

CMR: cardiac magnetic resonance; LGE: late gadolinium enhancement; FDG-PET-CT: fluorodeoxyglucose positron emission tomography integrated with computed tomography [2].

**Table 2:** Summary of the baseline characteristics of CS referrals.

	Patients (n = 51)
Age, years	58 ± 11
Male	31 (61)
Co-morbidities	
Hypertension	12 (24)
Heart failure	12 (24)
Hypercholesterolaemia	8 (16)
COPD/asthma	7 (14)
Ischaemic heart disease	6 (12)
Diabetes mellitus	5 (10)
Atrial fibrillation	4 (8)
CKD	2 (4)
Smoking (ex- or current)	1 (2)
Extra-cardiac sarcoidosis (ECS)	
Any evidence of ECS	29 (57%)
Biopsy confirmed ECS	15 (29%)
Medications	
Beta-blocker	27 (53)
SGLT-2 inhibitor	17 (33)
MRA	16 (31)
Sacubitril/Valsartan	13 (25)
ACE-inhibitor/ARB	12 (24)
Statin	20 (39)
Anti-platelet drugs	11 (22)
Anticoagulation	7 (14)
Loop diuretics	6 (12)

ACE: angiotensin converting enzyme; ARB: angiotensin receptor blocker; CKD: chronic kidney disease; COPD: chronic obstructive airways disease; MRA: mineralocorticoid receptor antagonist; and SGLT-2: Sodium-glucose co-transporter-2.

**Table 3:** Comparative diagnostic efficacy between the CS service and controls.

	CS Service Referrals (n = 51)	Controls (n = 24)	*p* Value
Age, years	58 ± 11	54 ± 14	0.296
Males	31 (61)	18 (75)	-
HRS criteria			
Positive	13 (25)	7 (29)	-
Clinical CS diagnosis made	13 (100)	2 (29)	-
Negative	38 (75)	17 (71)	-
Clinical CS diagnosis made	8 (21)	0 (0)	-
Endomyocardial biopsy	4 (8)	0 (0)	-
Positive	0 (0)	-	-

HRS: Heart Rhythm Society.

**Table 4:** HRS-negative patients adjudicated as having CS by specialist MDT.

Case	EMB	Radiological ECS	Histological ECS	Sustained VT	High-Degree AVB	FDG-PET Consistent with CS	CMR Consistent with CS
1	Not done	Yes	No	No	No	No	Yes
2	Not done	Yes	No	Yes	Yes	Yes	Yes
3	Negative	Yes	No	Yes	Yes	Yes	Yes
4	Not done	Yes	No	No	No	Yes	Yes
5	Negative	Yes	No	Yes	Yes	Yes	Yes
6	Not done	Yes	No	No	No	No	Yes
7	Not done	Yes	No	No	No	No	Yes
8	Negative	Yes	No	No	Yes	Yes	Yes

AVB: atrioventricular heart block; CS: cardiac sarcoidosis; ECS: extra-cardiac sarcoidosis; EMB: endomyocardial biopsy; FDG-PET: [18^F^]fluorodeoxyglucose positron emission tomography integrated with computed tomography; VT: ventricular tachycardia.

**Table 5:** Summary of LV dysfunction and ventricular arrhythmia treatments in CS patients.

	Patients with a Clinical CS Diagnosis (n = 21)
LV ejection fraction (%)	51 ± 11
LV systolic dysfunction (LVEF < 50%)	8 (38)
Received GDMT	7 (33)
Did not receive GDMT	1 (5) *
RV ejection fraction (%)	50 ± 12
LGE presence	20 (95)
ICD implantation	8 (38)
ICD indications	
High-grade atrioventricular heart block	5 (24)
Sustained ventricular tachycardia	2 (10)
LVEF < 35% despite GDMT	1 (5)

* This patient had concurrent severe aortic stenosis prohibiting establishment of optimal GDMT for heart failure. CS: cardiac sarcoidosis; GDMT: guideline directed medical therapy for heart failure; ICD: implantable cardioverter defibrillator; LGE: late gadolinium enhancement; LV: left ventricular; LVEF: left ventricular ejection fraction; and RV: right ventricular.

## Data Availability

The study data cannot be publicly shared due to the presence of patient clinical information. Reasonable requests for an anonymised dataset can be sent to the corresponding author.
